# Strain‐specific quantification of *Wolbachia* density in subtropical Argentinean *Aedes albopictus*: effects of tissue location and longevity

**DOI:** 10.3389/finsc.2025.1655459

**Published:** 2025-09-30

**Authors:** Ailén Chuchuy, Marcela S. Rodriguero, M. Victoria Micieli

**Affiliations:** ^1^ Centro de Estudios Parasitológicos y de Vectores (CEPAVE-CCT-La Plata-CONICET-UNLP), La Plata, Argentina; ^2^ Instituto de Ecología, Genética y Evolución (IEGEBA), CONICET—Universidad de Buenos Aires, Buenos Aires, Argentina

**Keywords:** Asian tiger mosquito, *Wolbachia*, quantitative PCR, Arbovirus, biocontrol, longevity, bacterial density

## Abstract

The intracellular bacterium *Wolbachia pipientis* has emerged as a promising tool for controlling mosquito-borne diseases; however, key aspects of its biology remain insufficiently understood, particularly how *Wolbachia* influences vector competence for certain arboviruses. The main factors implicated are the activation of mosquito antiviral pathways and competition for cellular resources at the viral replication site. Transinfection of *Wolbachia* strains into vector populations has proven to be an effective strategy for controlling arboviral diseases. Here, we investigate the within-host density and tissue distribution of two naturally occurring *Wolbachia* strains—*w*AlbA and *w*AlbB—n *Aedes albopictus* from Argentina, where infection patterns diverge from those observed globally. Using quantitative PCR, we assessed symbiont density in ovarian (*n* = 5) and somatic tissues (*n* = 5) of adult females, and in adult males across different ages: 0, 5 and 14 days post-emergence (*n* = 5 per age group). Our results reveal superinfection in ovaries (*w*AlbA + *w*AlbB) with similar densities (median relative density*_w_*_AlbA_ = 3.78 and median relative density*_w_*_AlbB_ = 3.31), but only *w*AlbB was consistently detected in somatic tissues (median relative density*_w_*_AlbB_ = 0.41), suggesting tissue-specific distribution of strains. Additionally, *w*AlbB density in males remained stable throughout the adult lifespan (median relative density_Time0_ = 0.83; median relative density_time 5_ = 1.98; median relative density_time 14_ = 0.66). These findings support the hypothesis that *Wolbachia* somatic localization is strain-specific and may be under evolutionary selection, with implications for vertical transmission and host fitness. By advancing our understanding of *Wolbachia* density dynamics in a natural mosquito vector population, this study contributes critical baseline data to inform and optimize *Wolbachia*-based biocontrol strategies in regions at risk of arboviral outbreaks. Because the *w*AlbB strain from *Ae. albopictus* is widely used in replacement techniques, any knowledge of its behavior in natural host populations is valuable.

## 1 Introduction

The recent emergence and re-emergence of mosquito-borne diseases (MBD) like yellow fever (YF), dengue fever (DF), chikungunya fever (CHIKF) and Zika disease (ZVD) is a cause for international concern. While DF remains the world’s most prevalent arboviral disease with tens of millions of cases annually and severe outbreaks in the Americas, YFV persists in endemic foci with high fatality among severe cases, CHIKF continues to cause regional outbreaks with occasional severe neurological sequelae, and ZVD circulates at lower levels globally but poses a persistent threat due to its teratogenic potential (1–5). Changes in climate and anthropogenic factors (e.g., land-use transformations and the large-scale movement of people, animals, and goods) are altering environmental conditions. These changes can indirectly affect the transmission and geographical distribution of MBD by facilitating the spread and redistribution of disease vectors across regions (6, 7).

Current approaches to managing MBD primarily focus on reducing populations of both immature and adult mosquitoes through insecticide application and community-based efforts to eliminate breeding habitats (8, 9). However, despite substantial resource investment, long-term reductions in mosquito densities remain challenging, with insecticide resistance being one of the main contributing factors (10), and seasonal outbreaks continue to occur (11, 12). This highlights a widely acknowledged need for innovative, cost-effective, and efficient tools to control arboviruses (13, 14). The limited success of conventional control methods has driven the exploration of innovative entomological strategies. The reproductive parasite *Wolbachia pipientis* Hertig, 1936 (Rickettsiales: Rickettsiaceae) (hereafter, *Wolbachia*), an obligatory intracellular and maternally inherited bacterium found in many arthropod species, sounds as a promising environmentally friendly weapon against MBD (15). Its ability to invade and maintain itself through manipulation of its host reproduction can be used to diminish mosquito population levels. Laying in the induced cytoplasmic incompatibility (CI) between uninfected females and infected males, both suppression and replacement with immune mosquito strategies have been proposed (16). While the concept of using the intracellular bacterium *Wolbachia* to manage mosquito populations was introduced over five decades ago, its potential role in dengue control has only garnered significant attention in the last ten years (17). Field studies in Australia and Indonesia have shown that releasing *Wolbachia*-transinfected *Aedes aegypti* (Linnaeus, 1762) mosquitoes can lead to a significant and lasting decrease in the DF transmission (18). Notably, a cluster randomized trial in Yogyakarta, Indonesia, demonstrated a 77% reduction in dengue cases in areas treated with *Wolbachia* (19). In Brazil, pilot releases in Rio de Janeiro and Niterói successfully established *Wolbachia* in local mosquito populations. Subsequently, Niterói expanded the intervention city-wide, using a phased strategy that included community engagement, information campaigns, mosquito releases, and field monitoring of *Wolbachia* prevalence (20, 21). Something similar occurred in the Colombian cities of Bello, Medellín, and Itagüí (22, 23). However, further field data are needed to evaluate *Wolbachia*’s effectiveness against these viruses and to assess its broader public health impact, as some investigations from Colombia are suggesting (24). Thus, it is important to study other *Wolbachia* strains and mosquito vectors to facilitate the introduction of this technology in other at-risk countries. The Asian tiger mosquito *Aedes albopictus* (Skuse, 1894) (Diptera: Culicidae) has expanded significantly over the past three decades from its native range in Southeast Asia to regions across North and South America, Southern Europe, parts of Africa, and various islands in Oceania, where it is now well established (25, 26). This mosquito is a competent vector for at least 22 arboviruses, including CHIKF and all four DF serotypes (27, 28). Although *Ae. albopictus* generally plays a secondary role in the transmission of DF and CHIKF compared to *Ae. aegypti*—partly due to its lower vector competence (29)—it has been implicated in outbreaks of these viruses in locations such as Hawaii, Mauritius, Gabon, Madagascar, and La Réunion (30–32). Moreover, autochthonous transmission of both viruses in parts of Europe by this species (33–36) highlights its growing public health relevance on a global scale. In Argentina, *Ae. albopictus* was first detected in 1998 (37). Its geographic distribution is restricted to the northeastern provinces of Misiones (where it was initially observed) and Corrientes (where it arrived due to a recent range expansion) (38). While it has been considered a secondary vector in Argentina (39), the wide variation in transmission efficiency observed across the Americas (40) suggests a substantial risk of CHIKF and DF becoming established and spreading throughout tropical, subtropical, and even temperate areas of the continent.

**Figure 1:**
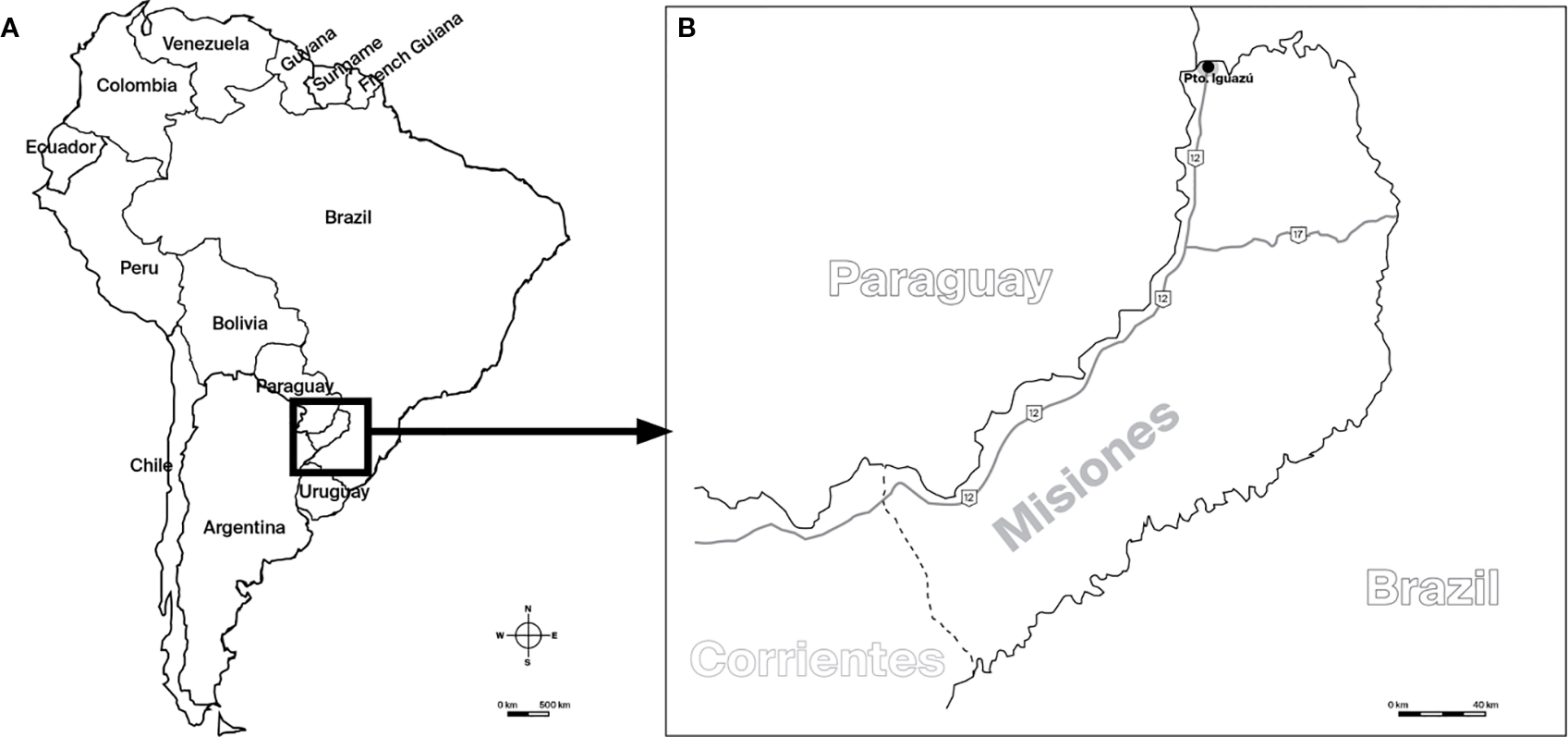
Map indicating the collection site for *Ae. albopictus*. **(A)** Regional map showing the relative position of Argentina within the continent. **(B)** Map of Argentina highlighting the sampling location in Puerto Iguazú. Maps were created using Adobe InDesign.

*Aedes albopictus* is naturally superinfected with two *Wolbachia* strains, identified as *w*AlbA and *w*AlbB (41), across nearly its entire global distribution (see **Figure 1** in (42)). A notable exception occurs in Argentina, where individuals have been found carrying only the *w*AlbB strain or lacking *Wolbachia* infection altogether (39). The relatively low vector competence observed in *Ae. albopictus* populations from the Argentinean subtropics—when compared to *Ae. aegypti*—may be influenced by the presence of *Wolbachia*. This hypothesis is supported by studies on *Ae. albopictus* from La Réunion Island, where the endosymbiont was shown to reduce DENV-2 dissemination and salivary gland infection (43). However, given potential differences in *Wolbachia* strains, densities, and environmental factors between regions, further investigation is warranted to clarify its role in modulating vector competence under local conditions.

Both *w*AlbA and *w*AlbB strains have been associated with increased resistance to arboviral infections in mosquitoes (43), with *w*AlbB typically reaching higher densities within the host (44). The density of the symbiont plays a critical role in the host–symbiont interaction, influencing not only the efficiency of maternal transmission but also the potential virulence of the symbiont itself (45, 46). Moreover, symbiont density has been identified as a key factor modulating immune function and antiviral responses in mosquitoes, alongside the genetic makeup of both the host and the *Wolbachia* strain (47, 48). Several studies have reported a positive correlation between *Wolbachia* density and the strength of antiviral protection in the mosquito host (47, 49, 50). Although the precise mechanisms by which *Wolbachia* influences vector competence remain to be fully elucidated, current evidence points to bacterial density as a major factor (47), as well as the bacterium’s localization in somatic tissues—outside the gonads—where it coexists with the pathogen (51). Given that *Wolbachia* strains differ in both tissue tropism and replication levels within their hosts (52–54), and that these parameters can vary between strains in superinfected individuals (46, 55–57), our study aims to quantify the density of *Wolbachia* strains in *Ae. albopictus* populations from Argentina. As a preliminary step to explore the symbiont’s potential influence on arbovirus transmission, we assessed variation in *Wolbachia* density according to tissue type in females. Additionally, we examined whether symbiont density changes with host age, in order to evaluate if this factor may underlie the previously reported infection polymorphism in Argentinean populations.

## 2 Materials and methods

### 2.1 Sample collection

Immature stages of *Ae. albopictus* were collected from a natural population at a go-kart track in Puerto Iguazú, Argentina (25°39’20”S–54°33’12”W) in February–March of 2019 (**Figure 1**), and reared to adulthood in the laboratory of the Centro de Investigaciones Ecológicas Subtropicales (CIES), at Puerto Iguazú. First instar larvae were separated in 1 L of dechlorinated water in a plastic flat tray with finely ground guinea pig food until pupation. Larval density was not quantified due to high and continuous larval mortality, which caused density to vary during this stage. The pupae were removed and individualized to plastic containers and provided with water and raisins in preparation for emergence. Following emergence adults were sexed and maintained in plastic vials containing cotton and moist filter paper, and fed raisins. Larvae and adults were kept in an incubator with a temperature cycle fluctuating between 21 °C and 34 °C, 60% of humidity and a photoperiod of 14:10 (L:D), simulating field conditions. The F0 generation was used in two simultaneous experiments: (i) comparison of *wAlbA* and *wAlbB* strain densities between ovaries and somatic tissues of females, and (ii) comparison of *wAlbB* density among mosquitos of different ages using males as a model. Both experiments were made at the CIES.

### 2.2 DNA extraction

DNA extraction was performed on ten adult individuals of *Ae. albopictus* (per experiment) using Chelex^®^ 100 resin (Bio-Rad, US). Each mosquito was placed in an Eppendorf tube containing 100 uL of 5% Chelex solution and 2 µL of Proteinase K (20 mg/mL; Promega, US). The mixture was incubated overnight at 56 °C for a period of 18 h. Proteinase K was then inactivated at 95 °C for 10 minutes. Subsequently, the sample was centrifuged at 14,000 rpm for 5 minutes, and the supernatant was transferred to a new tube and stored at −20 °C. DNA quality was assessed using a NanoDrop spectrophotometer (Thermo Fisher Scientific, USA).

### 2.3 Quantitative PCR assays

*Wolbachia* strain densities were investigated through quantitative PCR (hereafter, qPCR). Quantification of *wAlbA* and *wAlbB* strain densities was performed using the standard curve method. The wall surface protein gene (*wsp*) was used with strain-specific primers (**Table 1**). Quantification was normalized using the actin gene of *Ae. albopictus* as reference gene. These normalized values were used in comparative analyses (soma vs. ovary; and 0 days old males vs. 5days old males; 5days old males vs. 14 days old males). Target genes (*wsp* of *wAlbA* and *wAlbB*) and the reference gene (actin) were amplified from the same genomic DNA samples.

**Table 1:** List of primers used in qPCR assays, including primer ID, sequences (5′–3′), melting temperature (Tm), and GC content (%).

Organism	ID	Sequence (5’–3’)	Tm (°C)	% GC
*Ae. albopictus*	Act_F	CCTTCAACACACCGGCCATGTACG	65.3	58.3
*Ae. albopictus*	Act_R	TCAGATCGCGACCGGCCAAATC	64.0	59.1
*Wolbachia*	wspAlbA_F	CCAGTAGTTTCGCTATCAAAGTG	56.4	43.5
*Wolbachia*	wspAlbB_F	GTTGATCTCTTTAGTAGCTGATAC	53.8	37.5
*Wolbachia*	wspAlb_R	GTTGGTGTTGGTGTTGGTGCAG	61.5	54.5

Previously published primers (58–60) were first tested. Due to non-specific amplification in negative controls, new primers were designed and tested for specificity. Primer design was performed in Gene Runner V3.05 (61) with selection criteria as follows: amplicon size of 150–200 bp, GC content of 30–80%, and absence of secondary structures, which was checked with DNAMAN (62). The selected primers are shown in **Table 1**. To confirm annealing temperature and expected amplicon size (189 bp for actine gene, 194 bp for *w*AlbA *wsp* gene, and 213 bp for *w*AlbB *wsp* gene), endpoint PCR was performed under the following conditions: 94 °C for 15 s, 60 °C for 25 s, and 72 °C for 15 s (40 cycles). DNA from *Ae. albopictus* was used as template; DNase-free water was included as a negative control. PCRs were run on a Labnet Multigene thermal cycler (Thermo Fisher Scientific, US). Reaction volumes were 12.5 µl: 6.25 µl GoTaq Master Mix (Promega, US), 10 µM each primer (Macrogen, South Korea), 40 ng DNA, and 4.25 µl DNAse-free water. Amplicons were visualized on 2% agarose gels stained with ethidium bromide 0.4 µg/ml (Promega, US) under UV light. Two infected mosquitoes per *Wolbachia* strain were tested, and for the actin gene, one mosquito.

All quantitative PCR assays were conducted on a StepOne Plus instrument (Applied Biosystems, USA), using 96-well plates and MicroAmp™ adhesive seals (Thermo Fisher Scientific, USA), in a final volume of 20 μl following the design and reporting guidelines of Bustin et al. (63). Each reaction used 10 μl of a MasterMix with SYBR^®^ Green intercalating dye (Thermo Fisher Scientific, USA), 10 uM of oligonucleotides (Macrogen, South Korea), and 40 ng of total genomic DNA template.

Cycling conditions were 95 °C for 10 min, followed by 40 cycles of 15 sec at 95 °C and 1 min at 60 °C. Each plate included target and reference genes for each sample, with five biological replicates per comparison group, three technical replicates of each biological replicate and three technical replicates of negative controls per gene. Amplification quality was assessed by inspecting the amplification and melting temperature curves (see **Supplementary Material**). Cq values were averaged after verifying specificity via melting curves.

PCR efficiencies were calculated from standard curves (one per gene) generated using serial dilutions of a purified-PCR product (ranging from 10ng/µl and 0.001 ng/µl), with each dilution run in triplicate. Amplification efficiency (E) was calculated using the slope (m) of the linear regression line according to Applied Biosystems (2004): E = 10 (−1/m) – 1. Slopes ranging from –3.1 to –3.6 (corresponding to 90–110% efficiency) were considered acceptable. These efficiency values were used to validate the performance of the primer pairs, but not to calculate absolute quantities. Instead, relative quantification was performed using the comparative Cq method (ΔΔCt) (64). Normalized *Wolbachia* density in each sample was calculated as the ratio of *wsp* gene concentration (*w*AlbA or *w*AlbB) to *actin* gene amplification. These normalized values were used in statistical analyses. All analyses were conducted in R v4.1.0 (65) using integrated RStudio v1.0.153 environment (66).

### 2.4 Biological assays

#### 2.4.1 qPCR assay for comparison of bacterial density between soma and ovary

Five newly emerged females (biological replicates) were dissected immediately after emergence and sex determination to separate somatic and ovarian tissues under a light microscope. Each female was placed in a drop (ca. 20–30 ul) of PBS 1X on a microscope slide. Using fine forceps, the terminal abdominal segment was gently pulled to expose and remove the ovaries. The ovaries were rinsed in distilled water to prevent cross-contamination with somatic tissues, and the forceps were sterilized before handling the soma. Somatic and ovarian tissues were placed in separate Eppendorf tubes, and DNA was extracted as explained in subsection 2.2. DNA dilutions were prepared at a final concentration of 10 ng/µl and used in qPCR assays. The relative densities of *Wolbachia* strains *w*AlbA and *w*AlbB were compared between ovary and soma according to subsection 2.3. Relative densities obtained from this assay were compared using the Mann–Whitney U test using the rstatix (67) and ggpubr libraries (68). Box plots were generated using the base R function boxplot.

#### 2.4.2 qPCR assay to evaluate the variation in bacterial density of the *w*AlbB strain over male longevity

Because the *w*Mel strain may show reduced density and CI when *Ae. aegypti* larvae are reared at high temperatures (69–71), but the *w*AlbB strain proved to be much less susceptible to the effects of similar high rearing temperatures (70, 71), the latter might be well suited for population replacement in hot environments, given its ability to effectively block transmission of DF and other arboviruses (72). Thus, our efforts were primarily devoted to quantification of this strain. Additionally, attempts made to quantify the *w*AlbA strain yielded inconclusive results due to non-specific amplification. Thus, we proceeded to investigate only the density of the *w*AlbB strain.

The density of the *w*AlbB strain was measured in five males (biological replicates) at three time points: immediately after emergence, at 5 days post-emergence, and 14 days post-emergence. As explained before, larvae collected from the field were individually isolated in transparent plastic containers at the pupal stage. Adults were monitored daily. Five males were euthanized at each time point by cold exposure. DNA was extracted as described in subsection 2.2 and dilutions were prepared at 5 ng/µl for the actin gene and 50 ng/µl for the *wsp* gene of the *w*AlbB strain. The relative density was quantified according to subsection 2.3.

Comparisons of *w*AlbB density were performed between time points 0 and 1, and between 1 and 2. The results were analyzed with the Kruskal–Wallis test using libraries tidyverse (73). Box plots were generated using the base R function boxplot.

## 3 Results

### 3.1 qPCR assay for comparison of bacterial density in soma and ovary

Primer efficiency (Ef) resulted in Ef_wsp_*w*AlbB = 109.12% (R^2^ = 0.9911), Ef_wsp_*w*AlbA = 103.61% (R^2^ = 0.9884), and Ef_Actin = 117.83% (R^2^ = 0.9866). Although the actin primer showed an efficiency slightly exceeding the recommended range for the comparative Cq (ΔΔCt) method, no correction was applied. Given that all reactions were performed under identical conditions and involved comparative analyses within primer sets, we consider the relative quantification results to be reliable. However, this deviation is acknowledged as a limitation of the method. Melting showed three distinct peaks corresponding to each one *wsp*_*wAlbB* (87.5° C), *wsp*_*wAlbA* (80.1° C), and actin (78.6° C), confirming specific amplification for each target (see **Supplementary Material**).

**Figure 2:**
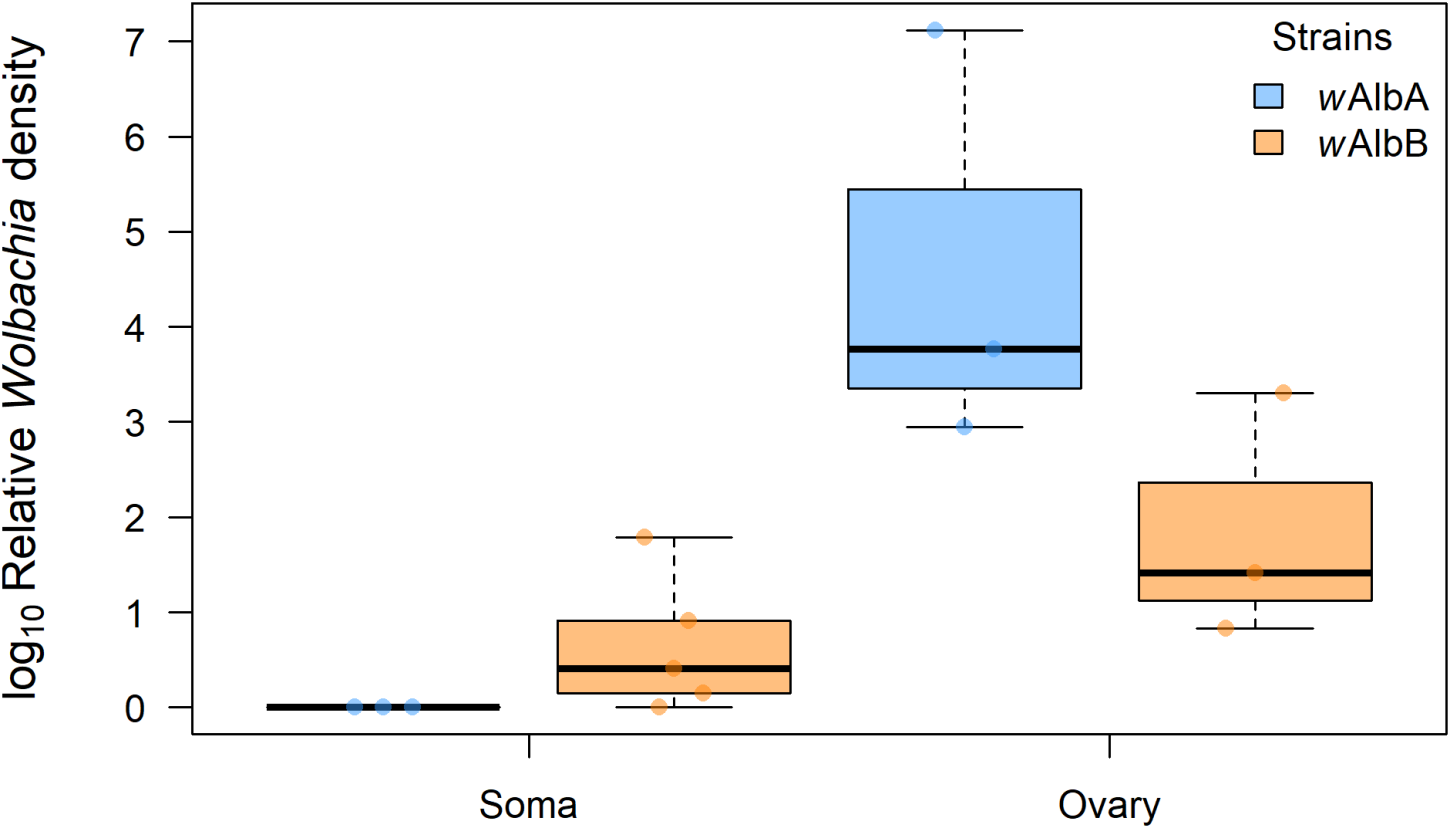
Relative densities of *Wolbachia* strains *w*AlbA (light blue) and *w*AlbB (orange) in ovaries and somatic tissues of *Ae. albopictus* females. Log10-transformed mean *Wolbachia* density is expressed as the ratio of the *Wolbachia* copy numbers of the gene *wsp* to the *Ae. albopictus* gene *Actin*, as estimated by qPCR on genomic DNA. A total of five biological replicates were used. Thick horizontal lines represent medians, box limits indicate first and third quartiles, whiskers represent interquartile range. Individual data points (jittered) are shown to illustrate the distribution of values.

Both *wAlbA* (median relative density = 3.78) and *wAlbB* (median relative density = 3.31) strains were detected in all five ovarian samples, indicating superinfection (**Figure 2**). No significant difference in density between *wAlbA* and *wAlbB* was observed in ovaries (Mann–Whitney U test, *p* > 0.05). In somatic tissues, *wAlbB* was detected in four of the five individuals (median relative density = 0.41), whereas *w*AlbA was not detected in any (**Figure 2**). A significant difference in bacterial density was observed between *w*AlbA and *w*AlbB in somatic tissues (Mann–Whitney U test, *p* < 0.05).

### 3.2 qPCR assay to evaluate changes in *wAlbB* density over male lifespan

For the assay evaluating the dynamics of *wAlbB* density over male lifespan, primer efficiencies were Ef_wspAlbB = 98.76% (R^2^ = 0.9920) and Ef_Actin = 111.55% (R^2^ = 0.9960). As these values were within or near the acceptable range for the comparative Cq (ΔΔCt) method, no correction was applied. However, we acknowledge that the actin primer exceeded the ideal efficiency range, which may have introduced some slight bias in the estimation. Melting temperatures were 79.03 °C for *wsp* (*wAlbB*) and 87.63 °C for actin. Melting curves showed specific amplification (see **Supplementary Material**).

**Figure 3:**
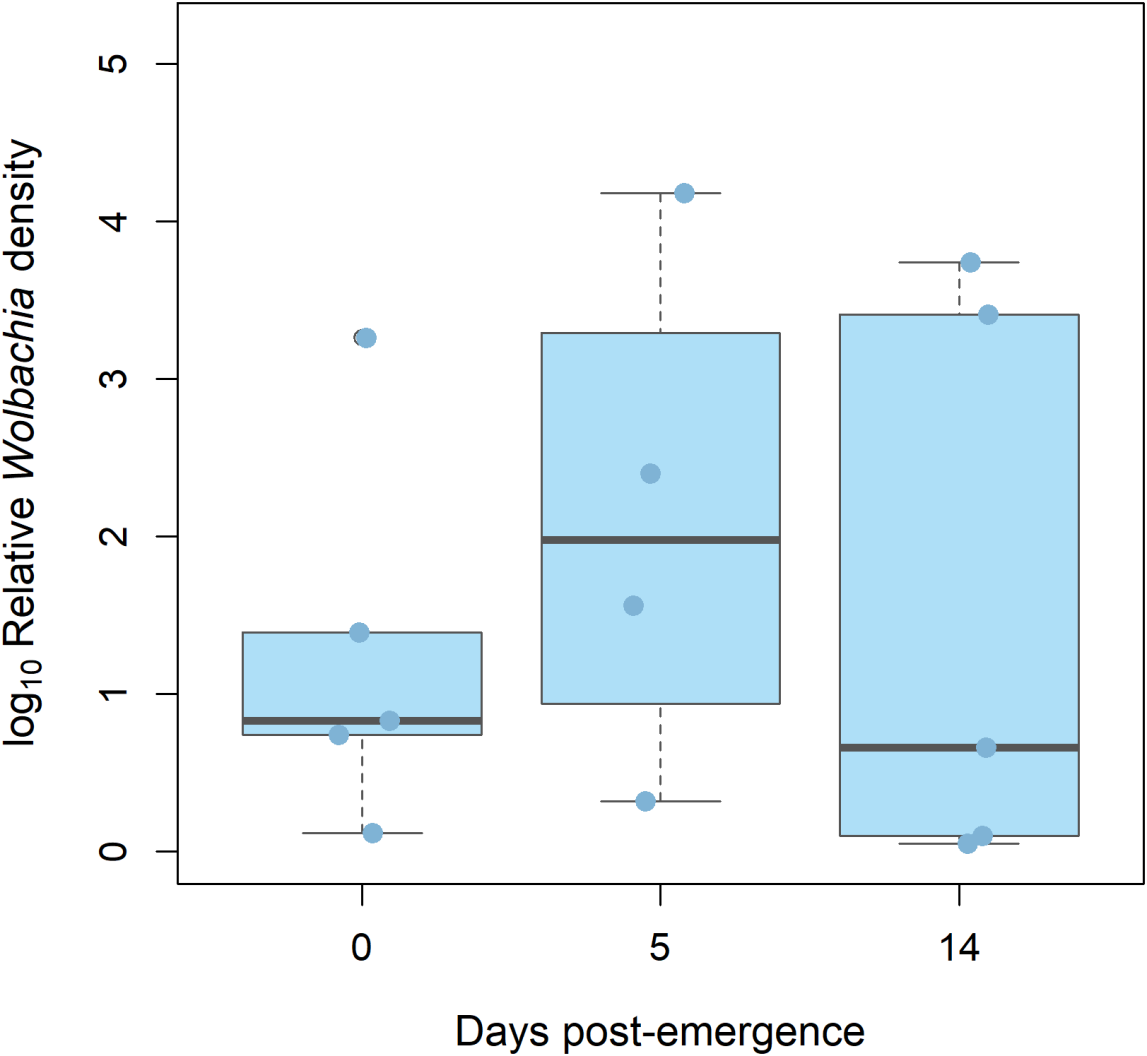
Relative densities of *Wolbachia* strain *w*AlbB (light blue) in 0, 5, and 14 days-old *Ae. Albopictus* males. Log10-transformed mean *Wolbachia* density is expressed as the ratio of the *Wolbachia* copy numbers of the gene *wsp* to the *Ae. albopictus* gene *Actin*, as estimated by qPCR on genomic DNA. A total of five biological replicates were used. Thick horizontal lines represent medians, box limits indicate first and third quartiles, whiskers represent interquartile range. Individual data points (jittered) are shown to illustrate the distribution of values.

A total of five males were analyzed for each time point. One sample from time = 14 days post-emergence was excluded due to ≥ 35 Cq value, in accordance with our pre-established threshold. No significant differences in *wAlbB* density were found between time = 0 days post-emergence (median relative density = 0.83) and 5 days post-emergence (median relative density = 1.98), nor between 5 and 14 (median relative density = 0.66) (Kruskal–Wallis test, *p* = 0.3362) (**Figure 3**).

Amplification of *wAlbA* was excluded due to poor melting curves, non-specific amplification, and high Cq values. One actin negative control showed a left-shifted curve, likely due to primer dimers, while the other showed no amplification. No such issues were observed in test samples.

## 4 Discussion

Bacterial density plays a critical role in all the phenotypes induced by *Wolbachia* in their hosts (74). Recent studies have reinforced earlier observations suggesting that, beyond its localization in the germline, somatic localization is a conserved feature of *Wolbachia* infection, indicating that it is not incidental but rather a key aspect of *Wolbachia* biology (51).

In this study, we examined the density of both *wAlbA* and *wAlbB* strains in somatic and ovarian tissues of *Ae. albopictus*. Both tissues were infected, with somatic tissues harboring only *wAlbB*, while ovaries displayed a double infection. The negative result in both tissues of a single sample may indicate that the infection is not fixed in this host population or could be due to a technical limitation. Increasing the sample size may help clarify this issue. Nevertheless, our finding is consistent with Dobson et al. (52), who reported that *Ae. albopictus* individuals infected solely with *wAlbA* lacked *Wolbachia* in somatic tissues, suggesting that *wAlbA* is restricted to reproductive tissues. Conversely, Zouache et al. (75) found both strains (*wAlbA* and *wAlbB*) in somatic tissues (salivary glands and gut) and ovaries of *Ae. albopictus* from Réunion Island. They also reported higher *Wolbachia* densities in ovaries than in somatic tissues, but did not observe significant differences between the two strains in either tissue type—a result that aligns with our findings in ovarian tissues.

Several studies have shown that the two strains differ significantly in their within-host densities, with *wAlbB* often reaching higher levels (44). This disparity may reflect differences in replication rates, with the *Wolbachia* strains from supergroup A (e.g., *wAlbA*) generally showing slower proliferation (56, 76). Moreover, each *Wolbachia* strain replicates independently, meaning that the growth rate of one strain is unaffected by the presence of the other. One possible explanation is that supergroup B strains may penetrate host cells more efficiently and replicate more rapidly in reproductive tissues than supergroup A strains (77). Possible cellular and molecular mechanisms that mediate differential tissue localization include variation in the expression of host receptors or cellular factors required for bacterial entry and replication, differential activation of immune pathways, or competition between strains for colonization niches (51, 75, 78, 79). Similar patterns of strain-specific tissue localization have been reported in other *Aedes* species, supporting the hypothesis that these differences are biologically driven rather than artifacts of our sampling approach (80).

The presence of *Wolbachia* in specific somatic tissues suggests that somatic tropism is a trait under selection, not a random byproduct of infection. Somatic localization may be maintained evolutionarily because it facilitates horizontal transmission within and between species, contributing to genetic diversity (51). Additionally, it may confer advantageous phenotypes on the host that promote vertical transmission through the germline (51), potentially increasing host fecundity or improving the vertical transmission efficiency of *Wolbachia* (52). From the host perspective, somatic infection may contribute to antiviral protection by interfering with viral replication in tissues critical for vector competence, thereby potentially increasing host survival and reproductive success (81). These interactions suggest that tissue-specific localization could be shaped by mutual evolutionary benefits. Given the relevance of somatic localization for biocontrol applications, it is important to rule out the possibility that the target *Wolbachia* genes are actually nuclear insertions of bacterial DNA fragments, as these would fail to generate the desired effects (82, 83).

We also investigated the dynamics of *wAlbB* density over the adult lifespan of male mosquitoes. Our results showed no significant variation in *wAlbB* density throughout adult male aging. This finding contrasts with that of Tortosa et al. (84), who observed a positive correlation between *wAlbB* density and age in males from Corsica and Réunion Island, but a negative correlation in males from Greece. They concluded that the association between *wAlbB* density and age may vary depending on the population of origin. In our study, however, these two variables appeared to be independent. Since both experimental designs were fairly similar, it is likely that the divergent results stem from intrinsic population factors. These may include differences in the genetic background of the host (such as variation in nuclear–*Wolbachia* interactions), local environmental adaptation, and historical selective pressures acting on both host and symbiont. For instance, Mejia et al. (85) showed that relative *Wolbachia* densities can be predictable across tissues and generations, but still vary depending on population origin. Furthermore, host genetic background has been shown to influence *Wolbachia*-mediated phenotypes: for example, *wMel* introgressed into different *Ae. aegypti* genetic backgrounds in Brazil and Vietnam produced differences in both mean and variance of dengue virus susceptibility (86). These studies support the idea that intrinsic variation among mosquito populations can significantly modulate *Wolbachia* dynamics and associated phenotypes.

Although this study did not quantify *Wolbachia* density in females, it is well established that bacterial density plays a crucial role in infection stability and the manifestation of *Wolbachia*-induced phenotypes in the host (74). In females, *Wolbachia* density may affect vector competence, while in males it could influence the strength of CI. This may help explain the low hatching rate observed in the Argentine *Ae. albopictus* population (see 39). Low bacterial densities could impair vertical transmission, leading to uninfected individuals, or in the case of co-infection, result in the stochastic loss of one of the strains. Such dynamics could underlie the infection polymorphism for *Wolbachia* infection observed in this host population (39). On the other hand, understanding strain density in females is particularly important, as they are the vectors. It is essential to assess density across the lifespan of adult females to determine whether antiviral activity remains constant and can be sustained throughout their life. This is especially relevant for a sustainable *Wolbachia*-based control strategy, in which the symbiont must be maintained across generations and vertical transmission reliably ensured.

We acknowledge that the relatively small sample size in our study is a limitation. Small sample sizes can reduce statistical power, making it more difficult to detect subtle differences and potentially increasing the likelihood of Type II errors. In addition, some data points were excluded due to technical issues in qPCR (e.g., failed amplification or outlier Cq values, since we discarded results with Cq ≥ 35), which further reduced the number of observations. We were also unable to include amplification of positive controls or sequencing of PCR products, which, to a certain extent, might limit the certainty of target specificity in our qPCR assays, although we partially verified the specificity of the reaction using melting curves. Despite these limitations, the observed trends were consistent across the analyzed samples, and the results provide valuable preliminary insights into tissue-specific *Wolbachia* densities.

In summary, the density of *Wolbachia* plays a critical role in shaping host–symbiont evolutionary interactions and enhancing the effectiveness of this bacterium as a biological control agent against insect pests and vector-borne diseases (16). Based on the results of this study, *w*AlbB emerges as a strong candidate for transinfection of native *Ae. aegypti* populations because of its somatic localization, which may enhance antiviral protection. If the high *w*AlbB density is consistently maintained throughout the female lifespan, we can expect both robust antiviral protection and reliable vertical transmission, thereby ensuring the sustainability of replacement biocontrol techniques.

Further experiments involving females—the sex that acts as arbovirus vectors—should assess *Wolbachia* strain density across the entire lifespan, which is relevant for antiviral activity; examine the dependence of bacterial density on temperature, as high temperatures can impair *Wolbachia* performance; and evaluate the influence of blood feeding on *Wolbachia* density, since arboviruses are acquired through blood meals, and it would be interesting to determine whether blood feeding promotes bacterial replication. Additionally, the main practical value of this work lies in the methodological information it provides for studying this strain, since we had to design several primer pairs and optimize real-time PCR conditions for multiple assays.

Given that the densities of *wAlbA* and *wAlbB* have been shown to differ across populations (87), it would be valuable to expand surveys to other locations of Argentina and explore how these variations correlate with superinfection patterns. Considering that *wAlbB* has demonstrated a strong potential to reduce dengue incidence in high-transmission areas (88, 89), further insights into this strain are of significant interest and importance. In order to validate the use of this strain in field settings in Argentina, the next steps would involve rearing native *Ae. aegypti*, as populations in Argentina are peculiar and replacement by foreign populations may be hindered by local adaptation and competition (90, 91), transinfecting them with the native *w*AlbB strain (the focus of our study), and conducting pre-release surveys.

## Data Availability

The raw data supporting the conclusions of this article will be made available by the authors, without undue reservation.
