# Supplementary material for: Strain‐specific quantification of Wolbachia density in subtropical Argentinean Aedes albopictus: effects of tissue location and longevity
Source: Front Insect Sci. 2025 Sep 30;5:1655459. doi: 10.3389/finsc.2025.1655459 (PMC12518245; doi:10.3389/finsc.2025.1655459)

Strain-specific quantification of *Wolbachia* density in subtropical Argentinean *Aedes albopictus*: effects of tissue location and longevity

Ailén Chuchuy[1†], Marcela S. Rodriguero[2*], M. Victoria Micieli[1]

[1]Centro de Estudios Parasitológicos y de Vectores (CEPAVE-CCT-La Plata-CONICET-UNLP), La Plata, Argentina
[2]Instituto de Ecología, Genética y Evolución (IEGEBA), CONICET - Universidad de Buenos Aires, Ciudad Autónoma de Buenos Aires, Argentina

[†]Present Address: Facultad de Turismo y Urbanismo - Universidad Nacional de San Luis, San Luis, Argentina

*** Correspondence**: Marcela S. Rodriguero – rodriguero@ege.fcen.uba.ar

Melting curves of the amplification products of the *wsp* genes from *wAlbB* (1) and *wAlbA* (2) strains, and the *actin* gene of *Aedes albopictus* (3), obtained from the bacterial density quantification assay in soma and ovary tissues. The negative control showed no amplification (4).

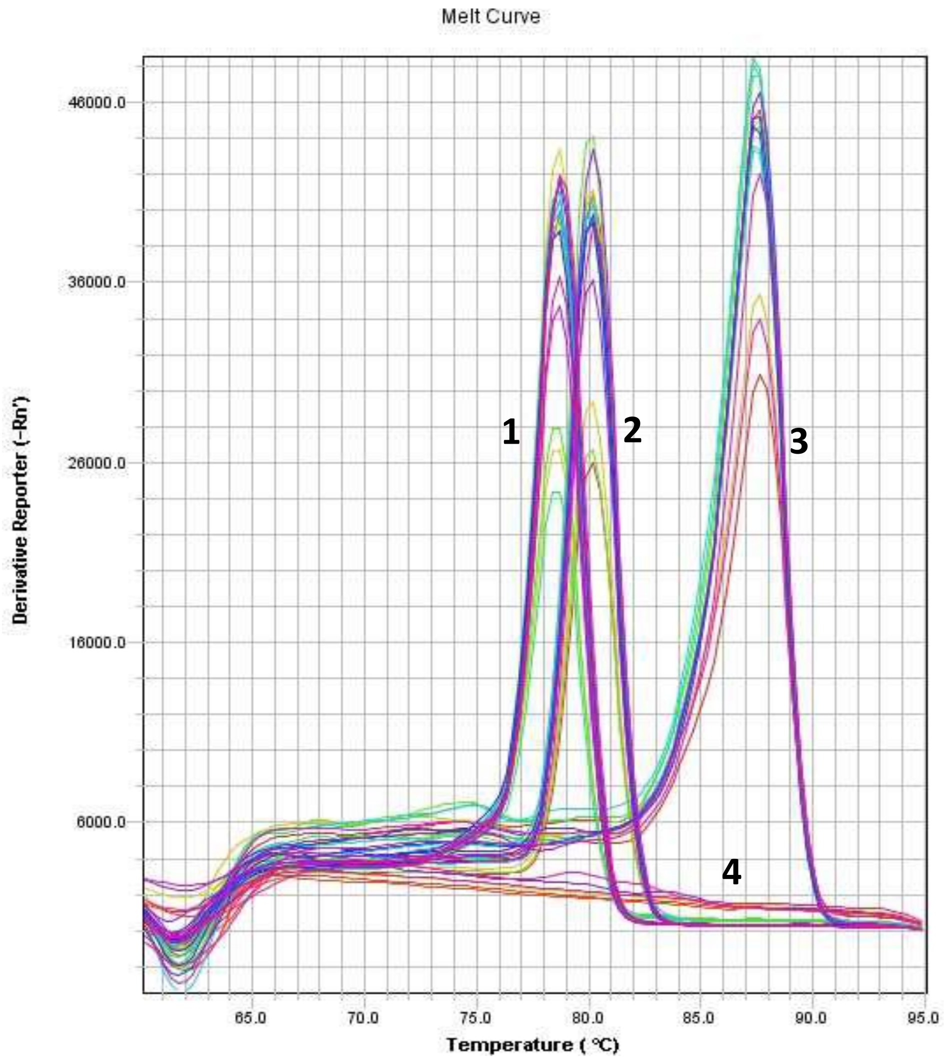

Melting curves of the amplification products of the *wsp* genes from the *w*AlbB strain (upper left) and the *actin* gene of *Aedes albopictus* (upper right), obtained from the male longevity assay. The negative control showed no amplification (4) with exception of one individual in the latter gene. This might be due to primer dimers,

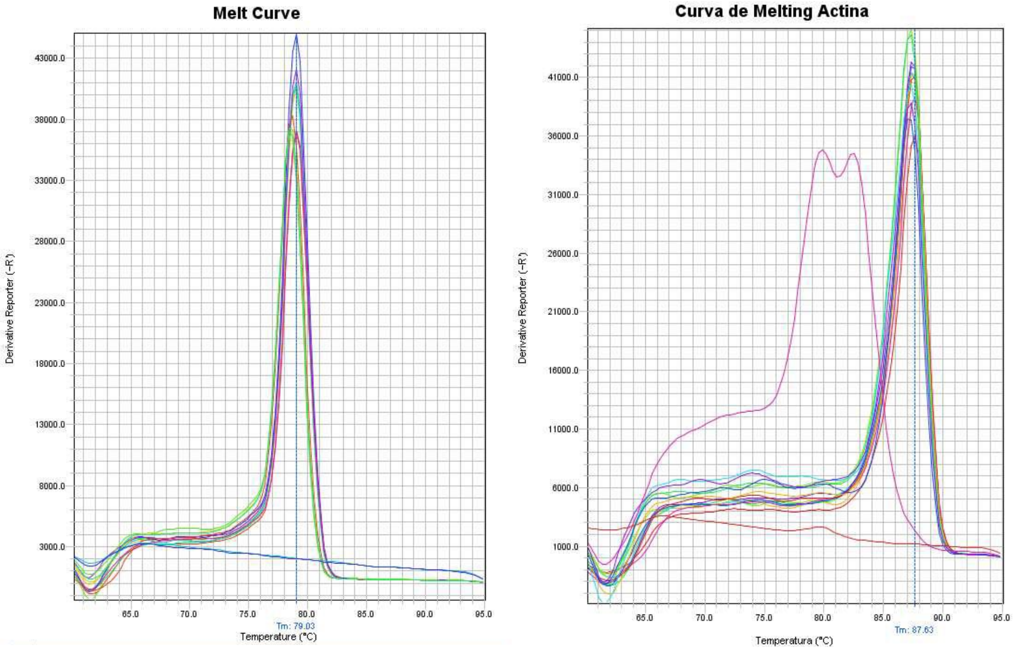

Supplement: Supplementary file 1 [file DataSheet1.pdf]
